# Plasma PlGF as a Potential Biomarker in Ramucirumab‐Based Second‐Line Therapy for Advanced Gastroesophageal Adenocarcinoma: Exploratory Biomarker Analysis from the Phase II Part of the RAMIRIS Trial

**DOI:** 10.1002/ijc.70567

**Published:** 2026-06-09

**Authors:** Jurek Hille, Sylvie Lorenzen, Claudia Pauligk, Victoria Gensch, Peter Thuss‐Patience, Eray Goekkurt, Thomas Ettrich, Florian Lordick, Carsten Bokemeyer, Christian Müller, Peter Reichardt, Martin Sökler, Daniel Pink, Stefan Probst, Thorsten O. Goetze, Salah E. Al‐Batran, Sonja Loges, Melanie Janning

**Affiliations:** ^1^ Department of Oncology, Hematology and Bone Marrow Transplantation With Section Pneumology University Medical Center Hamburg‐Eppendorf Hamburg Germany; ^2^ University Cancer Center Hamburg (UCCH), University Medical Center Hamburg‐Eppendorf Hamburg Germany; ^3^ Department of Medicine III TUM University Hospital, Technical University of Munich Munich Germany; ^4^ The Frankfurt Institute for Clinical Cancer Research IKF GmbH Frankfurt/Main Germany; ^5^ DKFZ‐Hector Cancer Institute at the University Medical Center Mannheim Mannheim Germany; ^6^ Division of Personalized Medical Oncology (A420) German Cancer Research Center (DKFZ), & Deutsches Zentrum für Lungenforschung (DZL) Heidelberg Germany; ^7^ Department of Personalized Oncology University Hospital Mannheim, Medical Faculty Mannheim University of Heidelberg Mannheim Germany; ^8^ Department of Hematology, Oncology and Cancer Immunology Charité‐University Medicine Berlin Berlin Germany; ^9^ Hematology‐Oncology Practice Eppendorf (HOPE) Hamburg Germany; ^10^ Department of Internal Medicine I Ulm University Medical Center Ulm Germany; ^11^ Department of Oncology, Gastroenterology, Hepatology, and Pulmonology University Cancer Center Leipzig (UCCL), Leipzig University Medical Center Leipzig Germany; ^12^ Department of Medical Oncology Evang. Kliniken Essen‐Mitte Essen Germany; ^13^ Sarcoma Center Berlin‐Brandenburg Helios Klinikum Berlin‐Buch Berlin Germany; ^14^ Oncology Spital Thun Thun Switzerland; ^15^ Second Department of Internal Medicine University Hospital Tübingen Tübingen Germany; ^16^ Department of Oncology and Palliative Care Helios Klinikum Bad Saarow Bad Saarow Germany; ^17^ Department of Internal Medicine C University Hospital Greifswald Greifswald Germany; ^18^ Department of Hematology, Oncology and Palliative Medicine Community Hospital Bielefeld Bielefeld Germany; ^19^ Krankenhaus Nordwest, University Cancer Center Frankfurt Frankfurt Germany; ^20^ Liquid Biopsy and Single Cell Analysis Group, Division of Personalized Medical Oncology (A420) German Cancer Research Center (DKFZ) & Deutsches Zentrum für Lungenforschung (DZL) Heidelberg Germany

**Keywords:** biomarker, gastric cancer, gastroesophageal cancer, placental growth factor, ramucirumab

## Abstract

Antiangiogenic treatment with ramucirumab (RAM) is a standard second‐line option in advanced gastric and gastroesophageal junction (GEJ) adenocarcinoma. However, reliable biomarkers are lacking. The phase II RAMIRIS trial compared RAM plus paclitaxel with RAM plus FOLFIRI (5‐fluororuracil, leucovorin and irinotecan) in this setting. We present the exploratory biomarker analysis evaluating placental growth factor (PlGF), carbonic anhydrase IX (CAIX), and tryptase. Plasma samples from 99 patients enrolled in RAMIRIS were collected at predefined timepoints (baseline, Cycle 2 Day 1, and Cycle 4 Day 1). PlGF, CAIX, and tryptase were quantified by ELISA. Associations with progression‐free survival (PFS) and overall survival (OS) were analyzed using dichotomized biomarker levels and Cox regression models. PlGF levels increased substantially under treatment, whereas CAIX showed a transient rise, followed by a slight decline, and tryptase remained stable. Elevated PlGF levels at baseline and early‐treatment (c2d1) were associated with shorter OS in univariate analysis (baseline HR_u_ = 1.75; *p* = 0.020; c2d1 HR_u_ = 1.69; *p* = 0.054). After multivariate adjustment, the association remained directionally consistent; although statistical support was retained only for c2d1 (baseline HR_m_ = 1.41, *p* = 0.198; c2d1 HR_m_ = 1.85, *p* = 0.030). CAIX and tryptase showed no consistent associations with survival. Elevated PlGF—particularly its early increase during RAM‐based therapy—was associated with shortened survival and may represent a dynamic marker of unfavorable prognosis in advanced gastric/GEJ adenocarcinoma. Given the exploratory nature of this analysis, these findings should be considered hypothesis‐generating and require validation in independent biomarker‐driven studies.

## Abstract (highlights)

What's new?

Antiangiogenic treatment with ramucirumab is a standard second‐line option in advanced gastric and gastroesophageal junction adenocarcinoma. However, reliable biomarkers are currently lacking. This exploratory biomarker analysis of the RAMIRIS trial suggests that elevated baseline and early on‐treatment levels of the pro‐angiogenic placental growth factor in patients with advanced gastric/gastroesophageal junction cancer are associated with shorter overall survival when treated with ramucirumab plus chemotherapy. Carbonic anhydrase IX (involved in hypoxia) and tryptase (mast cell–mediated vascular remodeling) showed no prognostic value. These hypothesis‐generating findings motivate prospective, pre‐planned biomarker studies towards informing patient stratification for anti‐angiogenic therapy in gastric and gastroesophageal cancers.

## Graphical abstract

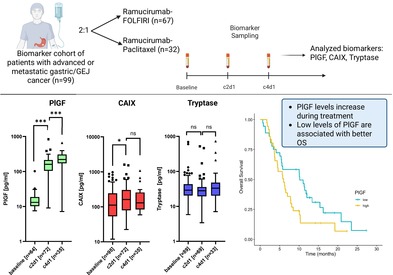

**Abbreviations**
CAIX: carbonic anhydrase IX
CI: confidence interval
ELISA: Enzyme‐linked Immunosorbent Assay
FOLFIRI: 5‐fluorouracil, leucovorin and irinotecan
GEJ: gastroesophageal junction
HR: hazard ratio
IQR: interquartile range
ITT: intention‐to‐treat
LOD: limit of detection
mCRC: metastasized colorectal cancer
OS: overall survival
PFS: progression‐free survival
PlGF: placental growth factor
RAM: ramucirumab
RAM‐FOLFIRI: ramucirumab plus 5‐fluororuracil, leucovorin and irinotecan
RAM‐Pac: ramucirumab plus paclitaxel
RECIST: Response Evaluation Criteria in Solid Tumors
VEGFR‐2: vascular endothelial growth factor 2 receptor

## 1 Introduction

In 2022, 968,784 (4.8%) new cases of gastric cancer and 511,054 (2.6%) new cases of oesophageal cancer were reported worldwide [1]. Gastric cancer ranks as the fifth most common cancer and the fourth leading cause of cancer‐related mortality worldwide, whereas oesophageal cancer ranks seventh and sixth, respectively [2].

Despite recent advances in the treatment of advanced gastro‐oesophageal cancer, particularly with the introduction of immunotherapy, second‐line treatment options remain limited. Ramucirumab (RAM) is a human IgG1 monoclonal antibody directed against vascular endothelial growth factor 2 receptor (VEGFR‐2). VEGFR2 is known to be over‐expressed in gastric cancer tissue [3]. The RAINBOW trial established RAM plus paclitaxel as a standard second‐line therapy in many countries [4, 5]. With taxanes moving also into earlier stages of treatment, the phase II RAMIRIS trial further investigated whether FOLFIRI (5‐fluororuracil, leucovorin and irinotecan) could serve as an effective combination partner for RAM compared to the standard RAM plus paclitaxel regimen in patients with advanced or metastatic gastro‐oesophageal AC. The study was stratified for previous docetaxel containing treatment. Although the primary endpoint (6 months overall survival (OS) rate of 65%) was not met, RAM‐ FOLFIRI showed promising efficacy in docetaxel‐pretreated patients with favorable response‐ and PFS rates and lower toxicity [6]. The phase III RAMIRIS trial is currently ongoing to further evaluate its efficacy.

Anti‐angiogenic treatment has been established in many different cancer types, but overall success was less than initial anticipated [7]. The discovery of biomarkers for anti‐angiogenic treatment has remained challenging. However, there is still a significant unmet need for translational research to stratify patients based on their likelihood of benefiting from anti‐angiogenic therapy.

This study reports an exploratory biomarker analysis of the phase II RAMIRIS trial. As the biomarker evaluation was not a pre‐specified endpoint of the protocol, all findings should be interpreted as hypothesis‐generating. The goal was to explore circulating biomarkers with potential prognostic value in patients receiving RAM‐based therapy. Based on our preclinical in vitro and in vivo data, we investigated carbonic anhydrase IX (CAIX), placental growth factor (PlGF), and tryptase in their soluble form, stemming from their mechanistic relevance to hypoxia (CAIX), pro‐angiogenic signaling (PlGF), and mast cell–mediated vascular remodeling (tryptase), respectively—all of which have been implicated in resistance or response to anti‐angiogenic treatment [8, 9, 10, 11, 12, 13].

## 2 Materials and Methods

### 2.1 Study Design and Participants

The phase II part of the RAMIRIS trial (EU Clinical Trials Register no: 2015‐00517124), including patient eligibility, trial design, randomization, dose administration, and statistical analysis has been published previously [6]. Briefly, RAMIRIS phase II was a prospective, multicenter, investigator‐initiated, randomized trial investigating the efficacy of FOLFIRI plus RAM versus the standard regimen of paclitaxel plus RAM in patients with advanced or metastatic gastroesophageal AC in second‐line therapy.

Patients were randomized in a 2:1 ratio to receive either RAM‐FOLFIRI or RAM‐Paclitaxel (RAM‐Pac). Patients in the experimental arm received irinotecan, 5‐FU, leucovorin on day 1 and 15 plus RAM 8 mg/kg iv on Day 1 and 15 of a 28‐day cycle. Patients in the control‐arm received paclitaxel on Day 1, 8, 15 and RAM on Day 1 and 15 of a 28‐day cycle until progression, intolerable toxicity or consent withdrawal but maximum for 1 year.

Randomization was stratified by prior docetaxel‐based therapy and by time to progression during or after first‐line therapy (≤ 3 months vs. > 3 months). Tumor assessment was performed according to Response Evaluation Criteria in Solid Tumors (RECIST), version 1.1 at baseline and every 2 months or until documentation of disease progression. After discontinuation of study medication, patients were followed for up to 1 year.

### 2.2 Sample Collection and Biomarker Assessment

Blood (EDTA) samples were collected at each center. EDTA tubes were centrifuged for 15 min at 1000 × g. Supernatant was frozen immediately at −20°C. Samples were obtained at baseline (Week 0), at cycle 2 Day 1 (Week 4), and at Cycle 4 Day 1 (Week 12). Frozen samples from each center were shipped to Hamburg for central biomarker analysis.

The potential biomarkers were analyzed with commercially available enzyme‐linked immunosorbent assay (ELISA) according to the manufacturer's instructions. CAIX (catalog no. DCA900), PlGF (catalog no. DPG00), both obtained from R&D (Minneapolis, USA), and tryptase (catalog no. SEB070) from Cloud Clone corp. (Houston, USA). All samples, standards, and controls were measured in duplicates. Protein concentrations were calculated by using a four‐parameter logistic (4PL) regression model.

### 2.3 Statistical Analysis

The biomarker population included all patients from the intention‐to‐treat (ITT) population with at least one biomarker measurement. The biomarker analyses were exploratory and not prespecified endpoints of the RAMIRIS trial.

To address non‐normal distribution, logarithmic transformation was applied to CAIX and tryptase levels, and square root transformation to PlGF (Figure S1A–F). Biomarker concentrations between timepoints were compared using the Mann–Whitney *U* test.

Associations between biomarker levels and clinical outcomes were analyzed after dichotomization at the median (“high” vs. “low”), with values below the lower detection limit assigned to the “low” group. Progression‐free survival (PFS) was defined as time from treatment initiation to disease progression or death, and OS as time from treatment initiation to death. Survival probabilities were estimated using Kaplan–Meier methods with 95% confidence intervals (CI).

For each biomarker, univariate and multivariate Cox proportional hazards models were applied to assess associations with OS and PFS. To account for potential immortal time bias when evaluating on‐treatment biomarker levels, landmark analyses were performed at predefined timepoints (c2d1 and, exploratorily, c4d1). In these analyses, only patients who were alive (for OS) and progression‐free (for PFS) at the landmark timepoint were included, and survival time was calculated from the landmark onward. Multivariate models included dichotomized biomarker level, treatment arm, age, gender, tumor location (GEJ vs. gastric), and the trial stratification factors (time to progression [≤ 3 months and > 3 months] and prior taxane treatment). *p* values were two‐sided and considered descriptive; no adjustment for multiple comparisons was performed due to the exploratory nature of the biomarker analyses.

Statistical analyses were performed using IBM SPSS Statistics 29.0.2.0, Kaplan–Meier curves were plotted with R 4.4.3.

## 3 Results

### 3.1 Patient Characteristics

**FIGURE 1:**
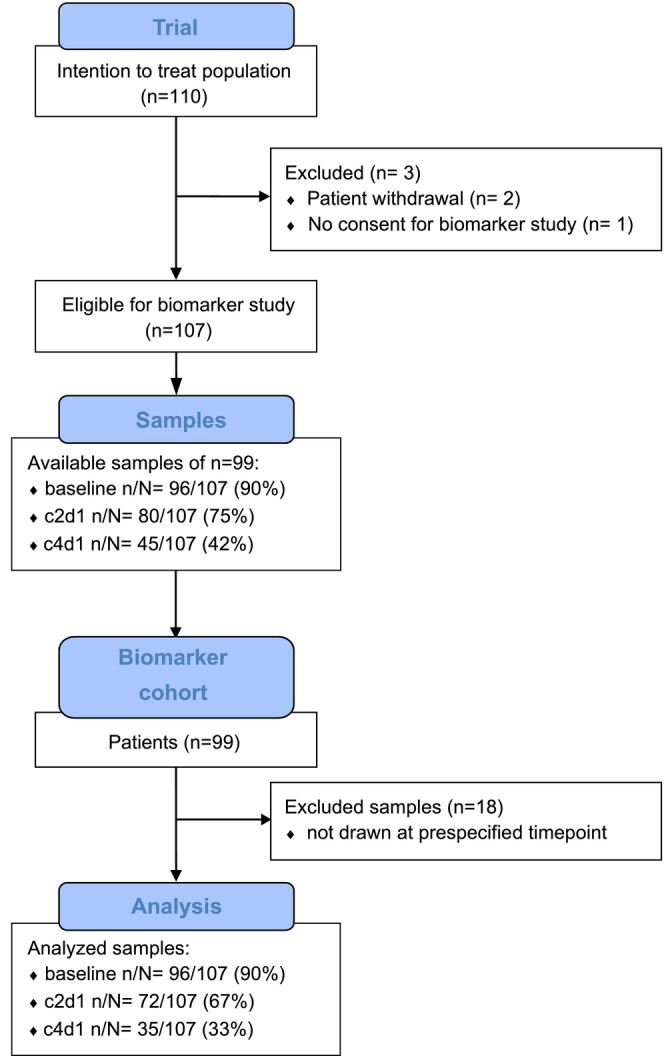
CONSORT flow diagram of biomarker project for the RAMIRIS trial.

A total of 111 patients were enrolled across 11 centers in this phase II trial. One patient, randomized to arm A, was excluded from the ITT population due to a protocol violation (HER2‐positive status without prior anti‐HER2‐targeted therapy, ITT = 110 patients). Three patients were excluded due to study withdrawal (*n* = 2) or lack of consent for biomarker analysis (*n* = 1). A total of 107 patients were eligible for the biomarker study and at least one blood sample was available from *n* = 99 patients (Biomarker cohort, Figure 1). In this cohort, *n* = 67 patients were allocated to arm A (RAM‐FOLFIRI) and 32 patients to arm B (RAM‐Pac, Table 1).

**TABLE 1:** Patient characteristics of biomarker cohort.

		RAM‐FOLFIRI	RAM‐Pac	Total
*n*		67	32	99
Age	Median	61	57	60
	< 60	29 (43%)	19 (59%)	48 (49%)
	60–69	30 (45%)	8 (25%)	38 (38%)
	≥ 70	8 (12%)	5 (16%)	13 (13%)
Gender	Male	44 (66%)	22 (69%)	66 (67%)
	Female	23 (34%)	10 (31%)	33 (33%)
ECOG	0	28 (42%)	17 (53%)	45 (46%)
	1	39 (58%)	15 (47%)	54 (55%)
	No	21 (31%)	12 (38%)	33 (33%)
Previous docetaxel therapy	Yes	46 (69%)	20 (63%)	66 (67%)
	Perioperative	19 (28%)	3 (9%)	22 (22%)
	1st line	31 (46%)	16 (50%)	47 (48%)
Primary tumor surgery	No	33 (49%)	20 (63%)	53 (54%)
	Yes	34 (51%)	12 (38%)	46 (47%)
Relevant comorbidities	No	4 (6%)	2 (6%)	6 (6%)
	Yes	63 (94%)	30 (94%)	93 (94%)
Histological type (Lauren)	Intestinal	24 (36%)	18 (56%)	42 (42%)
	Diffuse	22 (33%)	5 (16%)	27 (27%)
	Mixed	4 (6%)	1 (3%)	5 (5%)
	Unknown	13 (19%)	6 (19%)	19 (19%)
	Not applicable	4 (6%)	2 (6%)	6 (6%)
Number of metastatic sites	0	10 (15%)	2 (6%)	12 (12%)
	1–2	32 (48%)	21 (66%)	58 (53%)
	> 2	26 (36%)	11 (29%)	37 (34%)
Organ involvement	Liver	27 (40%)	15 (47%)	42 (42%)
	Lung	10 (15%)	10 (31%)	20 (20%)
	Peritoneum	23 (34%)	10 (31%)	33 (33%)
	Lymph nodes	34 (51%)	14 (44%)	48 (49%)
	Bone	14 (21%)	5 (16%)	19 (19%)
	Other	16 (24%)	5 (16%)	21 (21%)
Primary tumor localization	GEJ I	15 (22%)	7 (22%)	22 (22%)
	GEJ II	12 (18%)	6 (19%)	18 (18%)
	GEJ III	7 (10%)	4 (13%)	11 (11%)
	Gastric adenocarcinoma	32 (48%)	15 (47%)	47 (48%)
	Unknown	1 (2%)	0 (0%)	1 (1%)
Her2‐status	Negative	61 (91%)	28 (88%)	89 (90%)
	Positive	6 (9%)	4 (13%)	10 (10%)
Time to progression during 1st line therapy	≤ 3 months	42 (63%)	20 (63%)	62 (63%)
	> 3 months	25 (37%)	12 (38%)	37 (37%)

Overall, plasma samples were available for 96 patients (90% of eligible patients) at baseline, 80 patients (75%) at cycle 2, Day 1 (c2d1), and 45 patients (42%) at cycle 4, Day 1 (c4d1). The decline in available samples for later time points was primarily caused by patients developing disease progression. By the time of data‐cutoff (April 2020), 85 (77%) of 110 randomized patients had died, 55 (76%) patients in RAM‐FOLFIRI and 30 (79%) in the RAM‐Pac group. Only 9 out of 111 (8%) enrolled patients reached the maximum treatment period of 1 year.

As a note, a total of 18 plasma samples were excluded from biomarker analysis due to protocol deviations in blood collection timing (e.g., sampling at c2d8 instead of c2d1, Figure 1). Baseline characteristics of patients in the biomarker cohort are shown in Table 1. Overall, the characteristics were well‐balanced between treatment arms and comparable to the ITT population [6].

### 3.2 Biomarker Analysis

After ELISA was performed and concentrations were determined, only samples with biomarker level within the limits of detection (LOD) according to the manufacturer were included in biomarker concentration calculations. For CAIX, 95 samples were analyzed at baseline, 72 at c2d1, and 35 at c4d1. For tryptase, 89, 69, and 33 samples were analyzed, respectively. For PlGF, the number of analyzable samples was 64 at baseline, 72 at c2d1, and 35 at c4d1. For associations with clinical outcomes, patients with levels below the LOD were added to the “low” group, resulting in 93, 70, and 35 samples for tryptase and 96, 72, and 35 samples for PlGF, respectively, with no changes in sample numbers for CAIX.

### 3.3 Biomarker Concentrations at Baseline and Throughout Treatment

**FIGURE 2:**
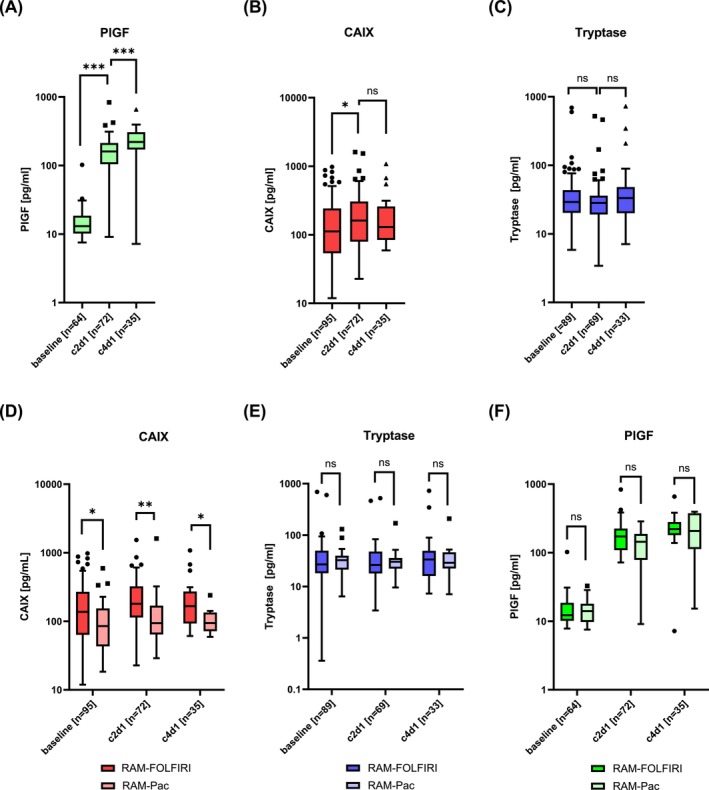
Plasma levels of PlGF, CAIX and Tryptase over the course of treatment (baseline, c2d1, c4d1). (A–C): Box plots for both treatment arms. (D–F): Box plots for in‐between comparison of treatment groups. Each box represents the interquartile range (IQR), with the horizontal line indicating the median. Whiskers extend to the smallest and largest values within 1.5 times the IQR from the lower and upper quartile, respectively. Individual points outside this range are plotted as outliers. Statistical comparisons were performed using Mann–Whitney tests.

The biomarkers—CAIX, tryptase, and PlGF—exhibited distinct patterns during treatment (Figure 2). PlGF showed a continuous increase during treatment: 13.1 pg/mL at baseline, rising to 160.6 pg/mL at c2d1 (*p* < 0.0001), and reaching 220.5 pg/mL at c4d1 (*p* = 0.0003; Figure 2A). CAIX concentration increased after the initiation of treatment (median 112.1 pg/mL at baseline, 161.6 pg/mL at c2d1, *p* = 0.045), followed by a decrease from c2d1 to c4d1 (129.5 pg/mL at c4d1, *p* = 0.664; Figure 2B). Tryptase levels remained relatively stable throughout treatment, with median values of 28.0 pg/mL at baseline, 28.4 pg/mL at c2d1, and 33.3 pg/mL at c4d1 (Figure 2C).

Next, we examined whether there was a difference in biomarker concentration between the two different treatment groups RAM‐FOLFIRI and RAM‐Pac. Patients in the RAM‐FOLFIRI arm had a higher concentration of CAIX compared to the RAM‐Pac group at every time point (Figure 2D). Conversely, there were no differences between groups for Tryptase or PlGF (Figure 2E,F).

### 3.4 Impact of Biomarkers on Survival Outcomes

Next, we wanted to test if biomarker levels were associated with outcome and could potentially serve as a biomarker for response. For this, patients were stratified into biomarker “high” (≥ median concentration) and “low” (< median concentration) subgroups for each time point. These subgroups were then analyzed for their association with PFS and OS, both for the overall cohort and within the treatment arms.

**FIGURE 3:**
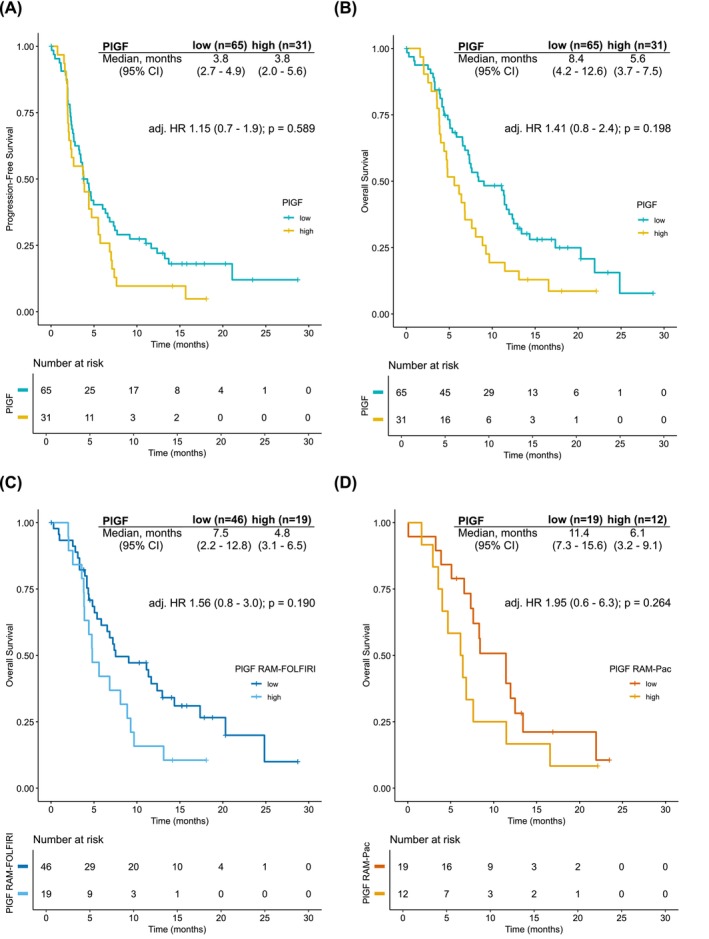
Kaplan–Meier survival curves stratified by PlGF expression levels at baseline. (A) PFS and (B) OS for all patients in the biomarker cohort. (C, D) OS stratified by treatment groups. Survival probabilities were estimated using the Kaplan–Meier method. Hazard ratios (HR) with 95% confidence intervals (CI) were calculated using Cox proportional hazards regression. Censored events are indicated by tick marks.

At baseline, there was no association of PlGF levels with PFS for the total cohort (Figure 3A, Table 2). However, a positive signal towards lower PlGF levels being associated with longer PFS in the RAM‐FOLFIRI arm at c2d1 was detected in univariate and multivariate analyses (HR_u_ 2.41, 95% CI 1.2–4.9, *p* = 0.013 and HR_m_ 2.23, 95% CI 1.1–4.6, *p* = 0.029).

**TABLE 2:** Association of dichotomized biomarker levels (low and high) with PFS and OS at different time points (baseline, c2d1, c4d1).

Biomarker	Timepoint	Group	*n*	Univariate cox‐regression PFS		Multivariate cox‐regression PFS		Univariate cox‐regression OS		Multivariate cox‐regression OS	
				Hazard ratio, (95% CI)	*p*	Hazard ratio, (95% CI)	*p*	Hazard ratio, (95% CI)	*p*	Hazard ratio, (95% CI)	*p*
PlGF	*Baseline*	All patients	96	1.42 (0.90–2.25)	0.136	1.15 (0.70–1.88)	0.589	1.75 (1.09–2.82)	0.020	1.41 (0.84–2.40)	0.198
		RAM‐FOLFIRI	65	1.30 (0.72–2.34)	0.380	1.06 (0.56–2.00)	0.889	1.81 (0.99–3.30)	0.054	1.56 (0.80–3.04)	0.190
		RAM‐Pac	31	1.56 (0.74–3.30)	0.240	1.36 (0.50–3.73)	0.551	1.69 (0.77–3.70)	0.192	1.95 (0.61–6.29)	0.264
	*c2d1*	All patients	72	1.44 (0.86–2.43)	0.167	1.52 (0.89–2.60)	0.129	1.69 (0.99–2.88)	0.054	1.85 (1.06–3.23)	0.030
		RAM‐FOLFIRI	49	2.41 (1.20–4.85)	0.013	2.23 (1.09–4.56)	0.029	2.54 (1.25–5.13)	0.010	2.44 (1.17–5.09)	0.018
		RAM‐Pac	23	0.55 (0.22–1.39)	0.205	1.36 (0.43–4.36)	0.600	0.79 (0.32–1.90)	0.593	1.63 (0.53–5.01)	0.397
	*c4d1*	All patients	35	1.10 (0.47–2.54)	0.832	1.08 (0.44–2.66)	0.859	1.37 (0.61–3.07)	0.445	1.48 (0.62–3.58)	0.380
		RAM‐FOLFIRI	25	2.06 (0.68–6.22)	0.199	2.44 (0.66–9.08)	0.184	2.70 (0.90–8.07)	0.076	2.52 (0.81–7.84)	0.111
		RAM‐Pac	10	0.32 (0.59–1.68)	0.176	Not performed^a^		0.46 (0.09–2.38)	0.354	Not performed^a^	
CAIX	*Baseline*	All patients	95	1.14 (0.73–1.76)	0.566	1.20 (0.74–1.92)	0.462	1.31 (0.83–2.08)	0.242	1.42 (0.86–2.35)	0.167
		RAM‐FOLFIRI	65	1.12 (0.64–1.95)	0.698	0.98 (0.55–1.73)	0.932	1.18 (0.66–2.11)	0.571	1.09 (0.61–2.00)	0.770
		RAM‐Pac	30	1.49 (0.67–3.30)	0.331	1.63 (0.65–4.09)	0.296	2.31 (0.94–5.71)	0.069	2.50 (0.90–6.90)	0.078
	*c2d1*	All patients	72	0.98 (0.58–1.64)	0.930	1.18 (0.60–2.31)	0.641	1.08 (0.64–1.84)	0.766	1.37 (0.73–2.59)	0.329
		RAM‐FOLFIRI	49	1.01 (0.52–1.98)	0.978	0.84 (0.36–1.94)	0.678	1.07 (0.55–2.08)	0.841	1.15 (0.55–2.39)	0.715
		RAM‐Pac	23	1.44 (0.54–3.83)	0.464	2.80 (0.71–11.07)	0.142	1.38 (0.52–3.70)	0.520	3.23 (0.75–13.82)	0.115
	*c4d1*	All patients	35	1.14 (0.49–2.65)	0.756	1.29 (0.48–3.50)	0.616	1.28 (0.56–2.92)	0.559	1.56 (0.58–4.15)	0.377
		RAM‐FOLFIRI	25	1.79 (0.56–5.74)	0.325	2.77 (0.70–11.01)	0.147	1.49 (0.52–4.25)	0.457	2.04 (0.59–7.06)	0.260
		RAM‐Pac	10	0.51 (0.10–2.68)	0.427	Not performed^a^		0.53 (0.06–4.55)	0.562	Not performed^a^	
Tryptase	*Baseline*	All patients	93	1.06 (0.68–1.65)	0.798	1.21 (0.76–1.92)	0.416	0.90 (0.57–1.43)	0.652	1.04 (0.63–1.69)	0.892
		RAM‐FOLFIRI	63	0.79 (0.45–1.38)	0.410	1.13 (0.61–2.11)	0.696	0.75 (0.42–1.33)	0.323	1.09 (0.57–2.07)	0.798
		RAM‐Pac	30	2.02 (0.92–4.40)	0.076	2.10 (0.87–5.08)	0.099	1.38 (0.62–3.05)	0.432	1.52 (0.65–3.55)	0.330
	*c2d1*	All patients	70	0.89 (0.53–1.50)	0.654	0.97 (0.54–1.74)	0.916	1.02 (0.60–1.73)	0.943	1.31 (0.72–2.39)	0.383
		RAM‐FOLFIRI	47	0.60 (0.31–1.18)	0.138	0.55 (0.24–1.30)	0.173	0.68 (0.34–1.36)	0.276	0.70 (0.28–1.75)	0.445
		RAM‐Pac	23	1.66 (0.66–4.16)	0.280	2.86 (0.66–12.42)	0.161	2.17 (0.86–5.46)	0.099	4.02 (0.93–17.25)	0.062
	*c4d1*	All patients	35	0.55 (0.24–1.28)	0.167	0.51 (0.21–1.26)	0.144	0.52 (0.23–1.21)	0.129	0.40 (0.15–1.12)	0.080
		RAM‐FOLFIRI	25	0.42 (0.14–1.23)	0.114	0.21 (0.05–0.96)	0.044	0.37 (0.13–1.08)	0.068	0.31 (0.09–1.08)	0.065
		RAM‐Pac	10	0.80 (0.18–3.48)	0.767	Not performed^a^		0.67 (0.13–3.53)	0.633	Not performeds^a^	

^a^ Multivariable Cox regression was not performed for the R‐Pac subgroup at c4d1 due to the very limited sample size (*n* = 10), precluding reliable model estimation.

Interestingly, the effects of PlGF were stronger towards OS. Patients with low levels of PlGF at baseline had a longer OS compared to those with high levels—median 8.4 versus 5.6 months at baseline (Figure 3B). A univariate cox regression demonstrated a directional association between lower PlGF and longer survival (HR_u_ 1.75, 95% CI 1.1–2.8, *p* = 0.020). In the multivariate model, this association remained but with wider CI (HR_m_ 1.41, 95% CI 0.8–2.4, *p* = 0.198, Table 2). While the effects appeared stronger for RAM‐FOLFIRI than in RAM‐Pac, the effect was overall less pronounced in the multivariate analysis (Figure 3 C + D, HR_u_ 1.81, 95% CI 1.0–3.3, *p* = 0.054; HR_m_ 1.56, 95% CI 0.8–3.0, *p* = 0.190, Table 2).

**FIGURE 4:**
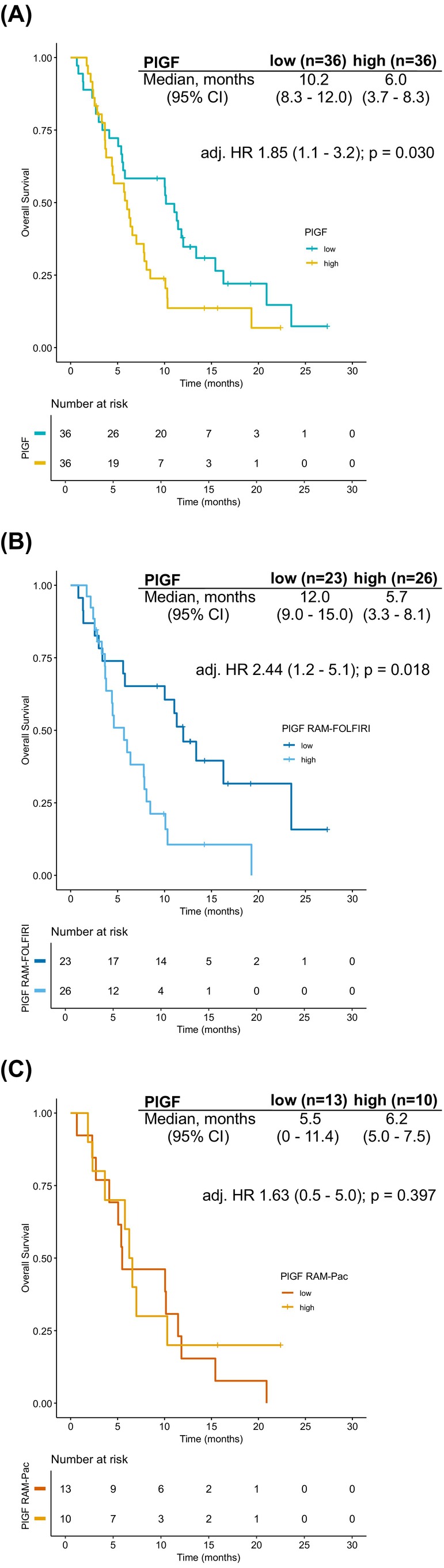
Kaplan–Meier survival curves stratified by PlGF expression levels at c2d1. (A) OS for all patients in the biomarker cohort. (B, C) OS stratified by treatment group. Survival probabilities were estimated using the Kaplan–Meier method. Hazard ratios (HR) with 95% confidence intervals (CI) were calculated using Cox proportional hazards regression. Censored events are indicated by tick marks.

However, for PlGF levels at c2d1, an association of PlGF with OS was observed for both uni‐ and multivariate analyses, with elevated PlGF levels continuing to correlate with worse outcomes. At c2d1, patients with low levels of PlGF demonstrated a median OS of 10.2 versus 6.0 months compared to those with high levels (HR_u_ 1.69, 95% CI 1.0–2.9, *p* = 0.054, Figure 4A). In the multivariate model, the estimate remained consistent (HR_m_ 1.85, 95% CI 1.1–3.2, *p* = 0.030). The survival benefit was also numerically stronger in the RAM‐FOLFIRI group compared to RAM‐Pac at c2d1 (Figure 4B,C). Multivariate analysis also confirmed a stronger association in RAM‐FOLFIRI versus RAM‐Pac (HR_m_ 2.44, 95% CI 1.2–5.1, *p* = 0.018 vs. HR_m_ 1.63, 95% CI 0.5–5.0, *p* = 0.397).

In multivariate analyses for CAIX and tryptase, no relevant associations with OS or PFS were observed (Figure S2A–D, Table 2).

Analyses for the later on‐treatment timepoint (c4d1) were also performed. However, due to substantial patient attrition at this stage—primarily driven by early disease progression—the number of evaluable patients was limited, resulting in reduced statistical power and potential selection bias. Accordingly, these results should be interpreted with caution. No consistent associations were observed across biomarkers (Table 2).

## 4 Discussion

In the phase II RAMIRIS trial, RAM‐FOLFIRI was not inferior in terms of PFS and OS to the standard regimen of RAM‐Pac in second‐line treatment for patients with advanced gastric/GEJ cancer and showed a potential benefit in patients who had received previous docetaxel treatment [6]. To explore biomarkers with potential prognostic value under RAM‐based therapy, we analyzed blood samples from 99 patients (89% of the ITT population) at different timepoints for their CAIX, tryptase, and PlGF concentrations. Our key findings were: (i) an increase in PlGF during RAM treatment, independent of the treatment group, and (ii) that patients with high PlGF levels had worse OS compared to those with low PlGF levels—an effect that was more pronounced for c2d1 PlGF than for baseline PlGF.

An increase in circulating PlGF during treatment has been consistently reported under VEGF/VEGFR pathway inhibition [14, 15, 16]. For instance, in the placebo‐controlled RAINBOW trial, PlGF levels increased exclusively in the RAM arm but not in the chemotherapy‐only control arm, supporting modulation by VEGFR‐2 blockade rather than by chemotherapy alone [16]. Similar PlGF elevations have been described in colorectal cancer trials evaluating bevacizumab or aflibercept‐based regimens [15]. Together, these data provide biological plausibility for the on‐treatment induction of PlGF under anti‐angiogenic therapy—in line with our data, although its clinical relevance remains uncertain.

The association between PlGF and outcome was less pronounced for PFS, which may be attributed to a general poor prognosis of patients with advanced gastric/GEJ cancer in second‐line setting, where rapid progression (< 4 months) was observed in both treatment arms. Such early progression is likely driven by aggressive tumor biology independent of anti‐angiogenic therapy, thereby blunting potential biomarker associations with PFS. Biologically, PlGF is also known to reflect unfavorable tumor biology, including immunosuppressive reprogramming of the tumor microenvironment (e.g., M2 macrophage polarization and impaired dendritic‐cell maturation and interruption of NK and dendritic crosstalk) [17, 18]. These mechanisms would be expected to exert a stronger influence on long‐term outcomes, consistent with the observed association with OS rather than short‐term disease control.

Furthermore, PFS is inherently more variable in this context, as assessments rely on predefined 8‐week imaging intervals. True progression events may thus have occurred between scans, limiting the sensitivity of PFS to detect biomarker‐related effects, particularly when PFS is short. OS, in contrast, is measured precisely and represents a more robust endpoint under these circumstances.

Notably, the association between PlGF and OS was stronger at c2d1 than at baseline. After multivariable adjustment, the baseline association was attenuated, whereas the on‐treatment effect remained robust. This pattern is biologically plausible, as PlGF upregulation likely reflects early adaptive angiogenic signaling in response to VEGF pathway inhibition, which would not yet be established prior to treatment initiation. These findings suggest that dynamic on‐treatment changes may provide more relevant prognostic information than baseline measurements alone.

A numerically stronger association between elevated PlGF levels and poorer OS was observed in the RAM‐FOLFIRI arm compared with the RAM‐Pac arm, particularly at c2d1. While this raises the possibility that the chemotherapy backbone may contribute to the observed effect, hazard ratios pointed in the same direction in both treatment arms. Given the limited sample size and the 2:1 randomization of the trial, differences in statistical power cannot be excluded, and regimen‐specific effects cannot be disentangled within this dataset. Accordingly, this observation should be regarded as hypothesis‐generating.

Previous investigations of PlGF and other angiogenic factors in gastric and colorectal cancer have yielded heterogeneous and partly inconsistent results, which need to be considered when interpreting our findings [14, 15, 16, 19, 20]. In advanced gastric/GEJ cancer, the placebo‐controlled RAINBOW trial demonstrated a marked on‐treatment increase in circulating PlGF levels exclusively in the RAM arm, consistent with a pharmacodynamic feedback mechanism under VEGFR‐2 inhibition [16]. However, no prognostic or predictive association of PlGF with clinical outcome was identified, and biomarker analyses primarily focused on baseline measurements, limiting direct comparability with our on‐treatment analyses. Biomarker evaluations in the REGARD trial did not include PlGF and did not identify a robust predictive angiogenic biomarker [20].

In metastatic colorectal cancer, the RAISE trial identified VEGF‐D as a potential predictive biomarker for RAM benefit, while PlGF was not assessed [19]. In contrast, the VELOUR trial evaluated aflibercept, a broader ligand trap targeting VEGF‐A, VEGF‐B and PlGF, and demonstrated clinical benefit irrespective of baseline PlGF levels, suggesting that broader angiogenic pathway inhibition may overcome compensatory mechanisms induced by selective VEGF/VEGFR blockade [15]. Beyond large randomized trials, a small retrospective tissue‐based study by Natsume et al. reported an association between high intratumoral PlGF expression and poorer outcomes in RAM‐treated gastric cancer patients [21]; however, these data are limited by small sample size, retrospective design, and differences in biological compartment and assay methodology, precluding direct comparison with circulating plasma biomarkers.

Taken together, the available literature underscores the absence of a validated predictive biomarker for anti‐angiogenic therapy in gastric/GEJ cancer and highlights the context‐dependent nature of PlGF associations. The novelty of our work lies in the prospective, serial assessment of circulating PlGF within the RAMIRIS trial. Nevertheless, despite biological plausibility supported by reproducible on‐treatment induction, PlGF should currently be regarded as an exploratory prognostic signal requiring independent validation in larger, prospectively designed biomarker‐driven studies before any clinical utility can be considered.

Several limitations of this analysis should be acknowledged. Biomarker assessments were not pre‐specified endpoints of the RAMIRIS trial and were conducted in a limited sample size. Importantly, the absence of a non‐RAM control arm precludes differentiation between prognostic and predictive effects. Previous studies evaluating PlGF and related angiogenic markers in gastric and colorectal cancer have yielded heterogeneous results, reflecting differences in tumor biology, treatment context, assay methodology, and biomarker timing.

In conclusion, our data demonstrates a reproducible on‐treatment increase in circulating PlGF and an association with poorer OS in RAM‐treated gastric/GEJ cancer patients. These findings support PlGF as an exploratory prognostic signal reflecting adaptive angiogenic responses under therapy. However, given the exploratory nature of the analysis and the lack of a non‐RAM comparator, PlGF cannot currently be considered clinically actionable. Independent validation in larger, prospectively designed studies with harmonized biomarker assessments is required before its potential role in guiding anti‐angiogenic therapy can be defined.

## 5 Conclusions

In this exploratory, non–pre‐specified biomarker analysis of the phase II RAMIRIS trial, elevated baseline and on‐treatment plasma PlGF levels were associated with shorter OS in patients with advanced gastric or GEJ adenocarcinoma receiving RAM‐based second‐line therapy. While causality cannot be inferred, the consistent on‐treatment increase of PlGF observed in this and other studies supports its potential role as a dynamic marker of adaptive angiogenic responses. In contrast, no consistent associations with clinical outcomes were observed for CAIX or tryptase. These results are hypothesis‐generating and warrant confirmation in prospectively designed, biomarker‐driven studies.

## Author Contributions

**Jurek Hille:** data curation, investigation, formal analysis, validation, writing – original draft, visualization, writing – review and editing. **Sylvie Lorenzen:** investigation, writing – review and editing. **Claudia Pauligk:** project administration, writing – review and editing. **Victoria Gensch:** investigation, project administration, writing – review and editing. **Peter Thuss‐Patience:** investigation, writing – review and editing. **Eray Goekkurt:** investigation, writing – review and editing. **Thomas Ettrich:** investigation, writing – review and editing. **Florian Lordick:** investigation, writing – review and editing. **Carsten Bokemeyer:** investigation, writing – review and editing. **Christian Müller:** investigation, writing – review and editing. **Peter Reichardt:** investigation, writing – review and editing. **Martin Sökler:** investigation, writing – review and editing. **Daniel Pink:** investigation, writing – review and editing. **Stefan Probst:** investigation, writing – review and editing. **Thorsten O. Goetze:** investigation, writing – review and editing. **Salah E. Al‐Batran:** investigation, writing – review and editing. **Sonja Loges:** conceptualization, methodology, supervision, resources, writing – review and editing, funding acquisition. **Melanie Janning:** methodology, validation, formal analysis, supervision, writing – original draft, writing – review and editing, investigation.

## Funding

The biomarker analysis was funded by an independent research grant provided by Eily Lilly and Company. This work was supported by the Margarete Clemens Stiftung, the E.W. Kuhlmann‐Stiftung, and the Hector II Stiftung.

## Ethics Statement

All patients provided written informed consent, and the study was conducted in compliance with the Declaration of Helsinki (2008). The study is registered in the EU Clinical Trials Register (EudraCT No. 2015‐005171‐24). The study protocol and all amendments were approved by the relevant institutional ethics committees at each participating center.

## Conflicts of Interest

C.B. declares consulting fees from advisory or data safety boards, honoraria for lectures and educational events, and travel support by AOK Germany, Astra Zeneca, Bayer Healthcare, BioNTech, Lindis Biotech, Sanofi Aventis GSO CRO, med update, Merck Serono, Roche Pharma, Dayichi‐Sankyo, all outside the submitted work; participation on advisory boards and leadership roles for the German Society of Hematology and Oncology (DGHO), the Hamburg Cancer Society (HKG), the German Cancer Society (DKG), the National Network of German Cancer Centers (CCC)/German Cancer Aid (DKH), and the Northern German Society of Internal Medicine (NWGIM), all outside the submitted work. C.M. reports consulting fees for advisory board from Lilly. D.P. reports research grants (institutional fees) from PharmaMar, BMS, Recordati, Roche, Deciphera, Boehringer‐Ingelheim and institutional fees from Roche (advisory role), PharmaMar (advisory role, lecture fee), Blueprint Medicines (lecture fee), Boehringer‐Ingelheim (advisory role, lecture fee), AstraZeneca (advisory role), Cogent (advisory role). F.L. reports personal fees for advisory board membership from Astellas, BeiGene, BMS, Daiichi Sankyo, MSD, PAGE and Servier; personal fees as an invited speaker from Art Tempi, Astellas, AstraZeneca, Incyte, Medscape, MedUpdate, Merck Serono, MSD, Servier and StreamedUp!; personal fees for expert testimony from BioNTech; personal fees for an editorial role as Editor‐in‐Chief from Elsevier and Springer Nature; institutional fees as a coordinating PI for BeiGene and the Frankfurt Institute of Clinical Cancer Research; institutional fees for research grants from AstraZeneca, BMS, Daiichi Sankyo and Gilead; non‐remunerated leadership role for the International Society for Diseases of the Esophagus; and non‐remunerated membership with the American Society of Clinical Oncology, German Cancer Society, German Society for Hematology and Oncology, German Society for Internal Medicine and the International Gastric Cancer Association. J.H. reports stock holdings in BioNTech and Novo Nordisk. M.J. reports received speakers' honoraria and or fees for advisory board participation from Roche, Amgen, AstraZeneca, Novartis, Takeda, MSD, Daiichi Sankyo and travel support from AstraZeneca and Takeda; participation in Daiichi Sankyo Endeavour Lung Program (2025–2027). P.R. reports honoraria from Deciphera, PharmaMar, Boehringer Ingelheim, Thermosome, Kabi, Merck. So.L. received personal fees as an invited speaker, consulting fees for advisory boards and travel support from Lilly, BerGenBio, Novartis, Boehringer Ingelheim, BMS, Roche, AstraZeneca, MSD, Merck, Novartis, Janssen, Takeda, Pfizer, Amgen, Bayer, Daiichi‐Sankyo, BeiGene, Apontis Pharma, Mirati, Sanofi; scientific research grants/IIT funding/industry‐sponsored trials (to institution) from Roche, BerGenBio, Lilly, AstraZeneca, Daiichi‐Sankyo, Janssen, IOVANCE, Gilead, Abbvie, PharmaMar, MSD. S.E.A.‐B. reports potential personal conflicts of interest for Frankfurter Institut für Klinische Krebsforschung IKF GmbH, Merck Sharp & Dohme, Eli Lilly Germany, Bristol‐Myers‐Squibb, MCI Deutschland GmbH and potential financial conflicts of interest for Celgene, Lilly Sanofi, German Cancer Aid (Krebshilfe), German Research Foundation, Federal Ministry of Education and Research of Germany, Roche, Vifor Pharma, Eurozyto, Immutep, Ipsen, Bristol‐Myers‐Squibb, MSD Sharp & Dohme, AstraZeneca. The other authors declare no conflicts of interest.

## Supporting information


**Figure S1:** Distribution of biomarker concentrations at baseline (pre‐treatment) before and after transformation. (A–D) logarithmic transformation of CAIX and tryptase. (E, F): square root transformation of PlGF.
**Figure S2:** Kaplan–Meier survival curves stratified by CAIX and Tryptase expression levels at baseline. PFS (A) and OS (B) for CAIX. PFS (C) and OS (D) for Tryptase. Survival probabilities were estimated using the Kaplan–Meier method. Hazard ratios (HR) with 95% confidence intervals (CI) were calculated using Cox proportional hazards regression. Censored observations are indicated by tick marks.

## Data Availability

The dataset supporting the conclusions of this article is included within the article and its additional files and are also available from the corresponding author on reasonable request.
