# Supplementary material for: Plasma PlGF as a Potential Biomarker in Ramucirumab‐Based Second‐Line Therapy for Advanced Gastroesophageal Adenocarcinoma: Exploratory Biomarker Analysis from the Phase II Part of the RAMIRIS Trial
Source: Int J Cancer. 2026 Jun 9;159(7):1817–28. doi: 10.1002/ijc.70567 (PMC13432362; doi:10.1002/ijc.70567)
Supplement: Supplementary file 1 — Figure S1: Distribution of biomarker concentrations at baseline (pre‐treatment) before and after transformation. (A–D) logarithmic transformation of CAIX and tryptase. (E, F): square root transformation of PlGF. Figure S2: Kaplan–Meier survival curves stratified by CAIX and Tryptase expression levels at baseline. PFS (A) and OS (B) for CAIX. PFS (C) and OS (D) for Tryptase. Survival probabilities were estimated using the Kaplan–Meier method. Hazard ratios (HR) with 95% confidence intervals (CI) were calculated using Cox proportional hazards regression. Censored observations are indicated by tick marks. [file IJC-159-1817-s001.pdf]

Supplementary Materials & Methods

Plasma PlGF as a Potential Biomarker in Ramucirumab-Based Second-Line Therapy for Advanced Gastroesophageal Adenocarcinoma: Exploratory Biomarker Analysis from the Phase II part of the RAMIRIS Trial

Jurek Hille, Sylvie Lorenzen, Claudia Pauligk, Victoria Gensch, Peter Thuss-Patience, Eray Goekkurt, Thomas Ettrich, Florian Lordick, Carsten Bokemeyer, Christian Müller, Peter Reichardt, Martin Sökler, Daniel Pink, Stefan Probst, Thorsten O. Goetze, Salah E. Al-Batran, Sonja Loges, Melanie Janning

Table of contents

Supplementary Figures

Figure S1 2

Figure S2 3

Figure S1

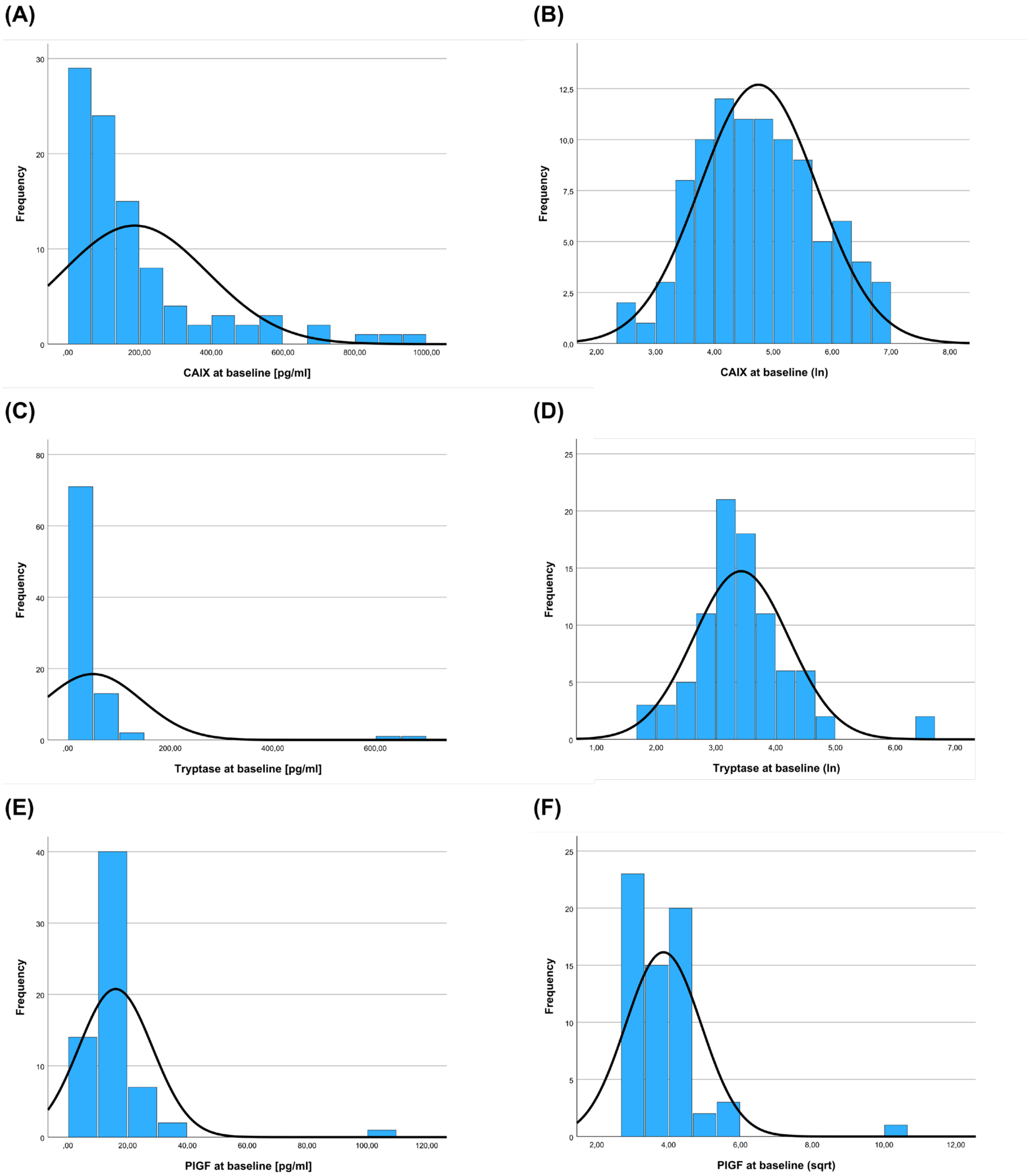

FIGURE S1 Distribution of biomarker concentrations at baseline (pre-treatment) before and after transformation. A-D: logarithmic transformation of CAIX and tryptase. E-F: square root transformation of PIGF.

Figure S2

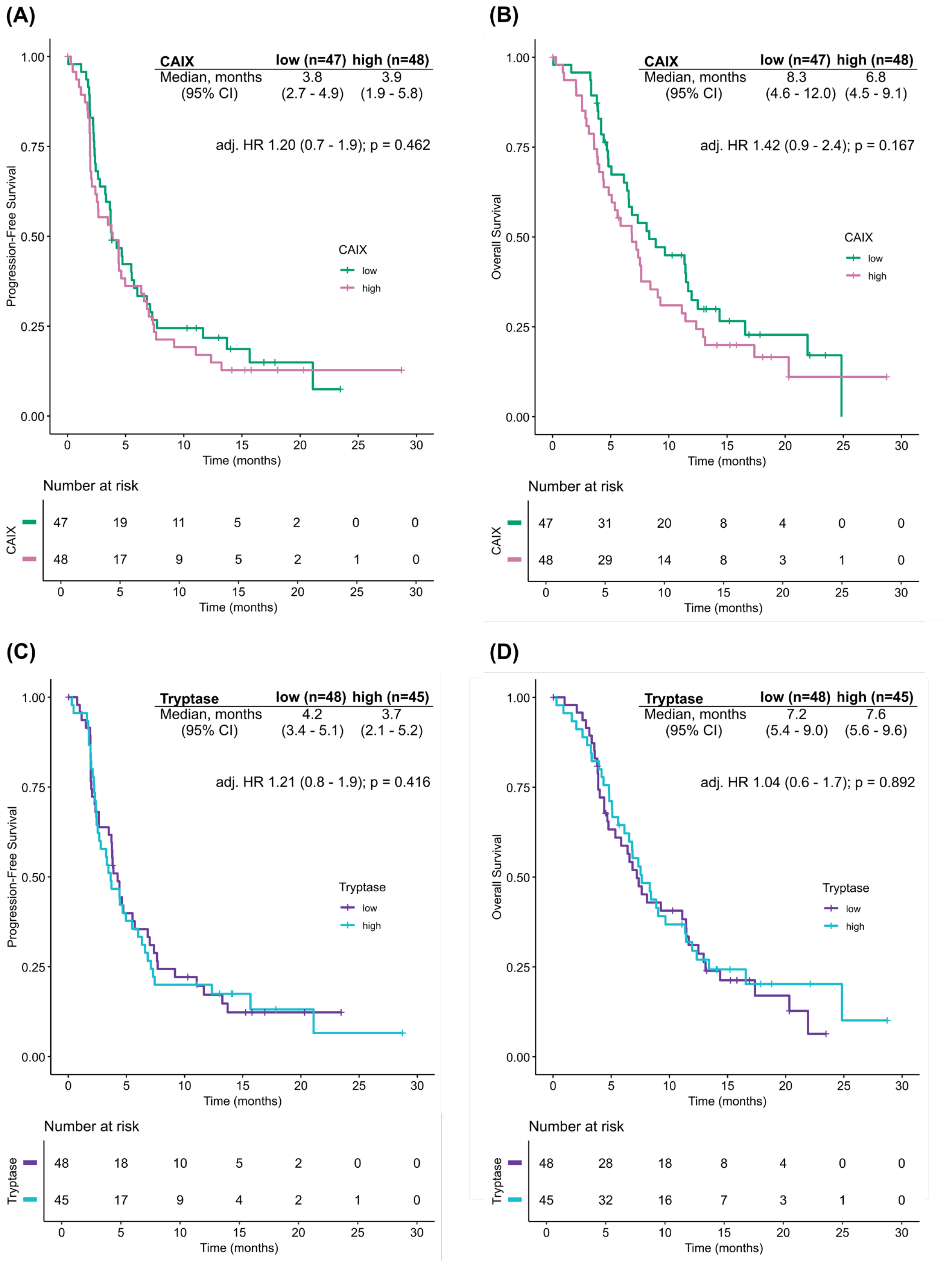

FIGURE S2 Kaplan–Meier survival curves stratified by CAIX and Tryptase expression levels at baseline. PFS (A) and OS (B) for CAIX. PFS (C) and OS (D) for Tryptase. Survival probabilities were estimated using the Kaplan–Meier method. Hazard ratios (HR) with 95% confidence intervals (CI) were

calculated using Cox proportional hazards regression. Censored observations are indicated by tick marks.
